# Consistency of option prices under bid–ask spreads

**DOI:** 10.1111/mafi.12230

**Published:** 2019-11-11

**Authors:** Stefan Gerhold, Ismail Cetin Gülüm

**Affiliations:** ^1^ Department of Financial and Actuarial Mathematics TU Wien Vienna Austria

**Keywords:** bid–ask spread, call option, martingale, peacock, Strassen's theorem, Transaction costs

## Abstract

Given a finite set of European call option prices on a single underlying, we want to know when there is a market model that is consistent with these prices. In contrast to previous studies, we allow models where the underlying trades at a bid–ask spread. The main question then is how large (in terms of a deterministic bound) this spread must be to explain the given prices. We fully solve this problem in the case of a single maturity, and give several partial results for multiple maturities. For the latter, our main mathematical tool is a recent result on approximation by peacocks.

## 1 INTRODUCTION

Calibrating martingales to given option prices is a central topic of mathematical finance, and it is thus a natural question which sets of option prices admit such a fit, and which do not. Note that we are not interested in *approximate* model calibration, but in the consistency of option prices, meaning arbitrage‐free models that fit the given prices *exactly*. Put differently, we want to detect arbitrage in given prices. We do not consider continuous call price surfaces, but restrict to the (practically more relevant) case of finitely many strikes and maturities. Therefore, consider a financial asset with finitely many European call options written on it. In a frictionless setting, the consistency problem is well understood: Carr and Madan (2005) assume that interest rates, dividends, and bid–ask spreads are zero, and derive necessary and sufficient conditions for the existence of arbitrage‐free models. Essentially, the given call prices must not admit calendar or butterfly arbitrage. Davis and Hobson (2007) include interest rates and dividends and give similar results. They also describe explicit arbitrage strategies, whenever arbitrage exists. Concurrent related work has been done by Buehler (2006). Going beyond existence, Carr and Cousot (2012) present practically appealing explicit constructions of calibrated martingales. More recently, Tavin (2015) considers options on multiple assets and studies the existence of arbitrage strategies in this setting. Spoida (2014) gives conditions for the consistency of a set of prices that contains not only vanillas, but also digital barrier options. See Henry‐Labordère, Obłój, Spoida, and Touzi (2016) for many related references.

As with virtually any result in mathematical finance, robustness with respect to market frictions is an important issue in assessing the practical appeal of these findings. Somewhat surprisingly, not much seems to be known about the consistency problem in this direction, the single exception being a paper by Cousot (2007). He allows positive bid–ask spreads on the options, but not on the underlying, and finds conditions on the prices that determine the existence of an arbitrage‐free model explaining them.

The novelty of our paper is that we allow a bid–ask spread on the underlying. Without any further assumptions on the size of this spread, it turns out that there is no connection between the quoted price of the underlying and those of the calls: Any strategy trying to exploit unreasonable prices can be made impossible by a sufficiently large bid–ask spread on the underlying (see Example 2.3 and Proposition 4.1). In this respect, the problem is *not* robust with respect to the introduction of a spread on the underlying. However, an arbitrarily large spread seems questionable, given that spreads are usually tight for liquid underlyings. We thus enunciate that the appropriate question is not “when are the given prices consistent,” but rather “how large a bid–ask spread on the underlying is needed to explain them?” Therefore, we put a bound ε≥0 on the spread of the discounted prices, and want to determine the values of ε that lead to a model explaining the given prices. We then refer to the call prices as ε‐consistent (with the absence of arbitrage). To define the payoff of the call options, we use an arbitrary reference price process that evolves within the bid–ask spread. We show (Proposition 2.5) that the consistency problem does not change dramatically if this reference process is the arithmetic average of the bid and ask prices of the underlying.

Recall that the main technical tool used in the papers (Carr & Madan, 2005; Cousot, 2007; Davis & Hobson, 2007) mentioned above to construct arbitrage‐free models is Strassen's (1965) theorem, or modifications thereof. In the financial context, this theorem shows the existence of martingale models for option prices that increase with maturity. The latter property breaks down if a spread on the underlying is allowed. We will therefore employ some results from our recent companion paper (Gerhold and Gülüm, 2019), which deals with variants of Strassen's theorem and approximating sequences of measures by peacocks (processes increasing with respect to the convex order).

We assume discrete trading times and finite probability spaces throughout; no gain in tractability or realism is to be expected by not doing so. In the case of a single maturity, we obtain simple explicit conditions that are equivalent to ε‐consistency (Theorem 3.1). The multiperiod problem, on the other hand, seems to be challenging. We provide two partial results: necessary (but presumably not sufficient) explicit conditions for ε‐consistency (Theorem 5.3), and sufficient semiexplicit conditions (Theorem 4.3). Here, by “semiexplicit,” we mean the following: Our consistency definition requires the existence of two sequences of measures, which are not “too far apart,” and one of which is a peacock. They correspond to a consistent price system, respectively, to a reference price that defines the option payoffs. Our result does not say anything about the existence of the reference price process, but contains explicit conditions for the existence of the peacock.

The structure of the paper is as follows. In Section 2, we describe our setting and give a precise formulation of our problem. Also, the significance of peacocks and approximating sequences of measures is explained. Then, in Section 3, we present necessary and sufficient conditions for the existence of arbitrage‐free models with bounded bid–ask spreads for a single maturity. Our main results on the multiperiod problem are contained in Section 4. There, we invoke the main result from Gerhold and Gülüm (2019). Necessary (but more explicit) conditions for multiple maturities are found in Section 5. Section 6 concludes.

## 2 THE CONSISTENCY PROBLEM UNDER BID–ASK SPREADS

Our time index set will be T={0,⋯,T}, where 1≤T∈N, and 0 means today. By a slight abuse of terminology, we will call the integers in T “maturities” and not “indices of maturities.” We write $T^{*}=\left\{1,\cdots ,T\right\}$ for the set of positive times in T. Whenever we talk about “the given prices” or similarly, we mean the following data:

(1) $$\begin{array}{cc} & A\;\text{positive}\;\mathrm{deterministic}\;\text{bank}\;\text{account}\;{\left(B\left(t\right)\right)}_{t\in T}\;\text{with}\;B\left(0\right)=1, \end{array}$$

(2) $$\begin{array}{cc} & \text{strikes}\;0<K_{t,1}<K_{t,2}<\cdots <K_{t,N_{t}},\;N_{t}\geq 1,\;t\in T^{*}, \end{array}$$

(3) $$\begin{array}{cc} & \text{corresponding}\;\text{call}\;\text{option}\;\text{bid}\;\text{and}\;\text{ask}\;\text{prices}\;\text{(at}\;\text{time}\;\text{zero)}\\ & \;0<{\underline{r}}_{t,i},\;\text{respectively,}\;0<{\bar{r}}_{t,i},\;1\leq i\leq N_{t},\;t\in T^{*}, \end{array}$$

(4) $$\begin{array}{cc} & \text{and}\;\text{the}\;\text{current}\;\text{bid}\;\text{and}\;\text{ask}\;\text{price}\;\text{of}\;\text{the}\;\text{underlying}\;0<{\underline{S}}_{0}\leq {\bar{S}}_{0}. \end{array}$$

We write $D\left(t\right)=B{\left(t\right)}^{-1}$ for the time zero price of a zero‐coupon bond maturing at *t*, and $k_{t,i}=D\left(t\right)K_{t,i}$ for the discounted strikes. The symbol $C_{t}\left(K\right)$ denotes a call option with maturity *t* and strike *K*.

In the presence of a bid–ask spread on the underlying, it is not obvious how to define the payoff of an option; this issue seems to have been somewhat neglected in the transaction costs literature. Indeed, suppose that an agent holds a call option with strike $100, and that at maturity T=1 bid and ask are ${\underline{S}}_{1}=\$99$, respectively, ${\bar{S}}_{1}=\$101$. Then, the agent might wish to exercise the option to obtain a security for $99 instead of $100, or he may forfeit the option on the grounds that spending $100 would earn him a position whose liquidation value is only $99. The exercise decision cannot be nailed down without making further assumptions. In practice, the quoted ticker price of the underlying is the last price at which an actual transaction has occurred. This price then triggers cash‐settled options. However, this approach is not feasible in our setup, which does not include an order book.

In the literature on option pricing under transaction costs, it is usually assumed that the bid and ask of the underlying are constant multiples of a mid‐price (often assumed to be geometric Brownian motion). This mid‐price is then used as trigger to decide whether an option should be exercised, followed by physical delivery (Bichuch, 2014; Davis, Panas, & Zariphopoulou, 1993; Whalley & Wilmott, 1997). The assumption that such a constant‐proportion mid‐price triggers exercise seems to be rather ad hoc, though. To deal with this problem in a parsimonious way, we assume that call options are cash‐settled, using a reference price process $S^{C}$. This process evolves within the bid–ask spread. It is not a traded asset by itself, but just serves to fix the call option payoff ${\left(S_{t}^{C}-K\right)}^{+}$ for strike *K* and maturity *t*. This payoff is immediately transferred to the bank account without any costs.

Definition 2.1 A *model* consists of a finite probability space (Ω,F,P) with a discrete filtration ${\left(F_{t}\right)}_{t\in T}$ and three adapted stochastic processes $\underline{S}$, $\bar{S}$, and $S^{C}$, satisfying^1^

(5) $$0<{\underline{S}}_{t}\leq S_{t}^{C}\leq {\bar{S}}_{t},\;t\in T^{*}.$$

Clearly, ${\underline{S}}_{t}$ and ${\bar{S}}_{t}$ denote the bid, respectively, ask price of the underlying at time *t*. Note that, in our terminology, the initial bid and ask are part of the given prices (see (4)), and thus the processes in Definition 2.1 are indexed by $T^{*}=\left\{1,\cdots ,T\right\}$ and not by T={0,⋯,T}.

As for the reference price process $S^{C}$, we do not insist on a specific definition (such as, e.g., $S^{C}=\frac{1}{2}\left(\underline{S}+\bar{S}\right)$), but allow *any* adapted process inside the bid–ask spread. We now give a definition for consistency of option prices, allowing for (arbitrarily large) bid–ask spreads on both the underlying and the options.

Definition 2.2 The prices (1)–(4) are *consistent with the absence of arbitrage*, if there is a model (in the sense of Definition 2.1) such that

- $E\left[{\left(D\left(t\right)S_{t}^{C}-k_{t,i}\right)}^{+}\right]\in \left[{\underline{r}}_{t,i},{\bar{r}}_{t,i}\right],\;1\leq i\leq N_{t},\;t\in T^{*}$.
- There is a process ${\left(S^{*}\right)}_{t\in T}$ such that ${\underline{S}}_{t}\leq S_{t}^{*}\leq {\bar{S}}_{t}$ for t∈T and such that ${\left(D\left(t\right)S_{t}^{*}\right)}_{t\in T}$ is a P‐martingale^2^ with respect to the filtration ${\left(F_{t}\right)}_{t\in T}$. The pair $\left(S^{*},P\right)$ is called a consistent price system.

The process $S^{*}$ is also called a shadow price. According to Kabanov and Stricker (2001) (see also Schachermayer, 2004), these requirements yield an arbitrage‐free model comprising bid and ask price processes for the underlying and each call option. Indeed, for the call with maturity *t* and strike $K_{t,i}$, one may take ${\left({\underline{r}}_{t,i}1_{\left\{s=0\right\}}+B\left(s\right)E\left[{\left(D\left(t\right)S_{t}^{C}-k_{t,i}\right)}^{+}|F_{s}\right]1_{\left\{s>0\right\}}\right)}_{s\in T}$ as bid price process (and similarly for the ask price), and ${\left(B\left(s\right)E\left[{\left(D\left(t\right)S_{t}^{C}-k_{t,i}\right)}^{+}|F_{s}\right]\right)}_{s\in T}$ as the process in the second part of Definition 2.2. We recommend section 1 of Schachermayer's (2017) recent book as an accessible introduction to the fundamental theorem of asset pricing under proportional transaction costs.

As mentioned in Section 1, if consistency is defined according to Definition 2.2, then there is no interplay between the current prices of the underlying and the options, which seems to make little sense. As an illustration, the following two‐period example shows how frictionless arbitrage strategies may fail in the presence of a sufficiently large spread; a general result is given in Proposition 4.1.

Example 2.3 Let c>0 be arbitrary. We set $k:=k_{1,1}=k_{2,1}=1$ and assume

$$B\left(1\right)=B\left(2\right)=1,\;{\underline{S}}_{0}={\bar{S}}_{0}=2,\;r_{1}:={\underline{r}}_{1,1}={\bar{r}}_{1,1}=c+1,\;r_{2}:={\underline{r}}_{2,1}\;={\bar{r}}_{2,1}=1.$$

Thus, $C_{1}\left(k\right)$ is “too expensive,” and without frictions, buying $C_{2}\left(k\right)-C_{1}\left(k\right)$ would be an arbitrage opportunity (upon selling one unit of stock if $C_{1}\left(k\right)$ expires in the money). In particular, the first condition from Corollary 4.2 in Davis and Hobson (2007) and equation (5) in Cousot (2007) are violated: they both state that $r_{1}\leq r_{2}$ is necessary for the absence of arbitrage strategies.

But with spreads we can choose *c* as large as we want and still the above prices would be consistent with no‐arbitrage. Indeed, we can define a deterministic model as follows:

$${\underline{S}}_{1}={\underline{S}}_{2}=2,\;{\bar{S}}_{1}=2c+2,\;{\bar{S}}_{2}=2,\;S^{C}=\frac{1}{2}\left(\underline{S}+\bar{S}\right).$$

Note that

$${\left(S_{2}^{C}-k\right)}^{+}=1\;\text{and}\;{\left(S_{1}^{C}-k\right)}^{+}=c+1.$$

This model is free of arbitrage (see Proposition 4.1). In particular, consider the portfolio $C_{2}\left(k\right)-C_{1}\left(k\right)$: the short call $-C_{1}\left(k\right)$ finishes in the money with payoff −(c+1). This cannot be compensated by going short in the stock, because its bid price stays at 2. The payoff at time t=2 of this strategy, with shorting the stock at time t=1, is

$${\left(S_{2}^{C}-k\right)}^{+}-{\left(S_{1}^{C}-k\right)}^{+}-\left({\bar{S}}_{2}-{\underline{S}}_{1}\right)=-c<0.$$

Our focus will thus be on a stronger notion of consistency, where the discounted spread on the underlying is bounded. Hence, our goal becomes to determine how large a spread is needed to explain given option prices.

Definition 2.4 Let ε≥0. Then the prices (1)–(4) are “ε‐consistent with the absence of arbitrage,” or simply “ε‐consistent,” if they are consistent (Definition 2.2) and the following conditions hold:

(6) $$\begin{array}{cc} {\bar{S}}_{t}-{\underline{S}}_{t} & \leq \varepsilon B(t),\;t\in T, \end{array}$$

(7) $$\begin{array}{cc} S_{t}^{C} & \geq \varepsilon B\left(t\right),\;t\in T^{*}. \end{array}$$

The bound (7) is an additional mild assumption on the reference price $S^{C}$, made for tractability, and makes sense given the actual size of market prices and spreads (recall that $\underline{S}\leq S^{C}$). With the same justification, in our main results on ε‐consistency we will assume that all discounted strikes $k_{t,i}$ are larger than ε. If ε=0 and the bid and ask prices in (3) and (4) agree, then we recover the frictionless consistency definition from Davis and Hobson (2007).

As mentioned above, we do not insist on any specific definition of the reference price $S^{C}$. However, it is not hard to show that choosing $S^{C}=\frac{1}{2}\left(\underline{S}+\bar{S}\right)$ yields almost the same notion of ε‐consistency.

Proposition 2.5 Let ε≥0 and assume that we are interested in arbitrage‐ free models where, in addition to the requirements of Definition 2.4, we have that

(8) $$\begin{array}{c} S_{t}^{C}=\frac{{\underline{S}}_{t}+{\bar{S}}_{t}}{2},\;t\in T^{*}. \end{array}$$

Let us then call the prices (1)–(4) *arithmetically* ε*‐consistent*. For ε≥0, the prices are arithmetically 2ε‐consistent if and only if they are ε‐consistent.

First, assume that there exists an arithmetically 2ε‐consistent model with corresponding stochastic processes ${\underline{S}}_{t},{\bar{S}}_{t},S_{t}^{C},S_{t}^{*}$. We define new bid and ask prices ${\underline{S}}_{t}^{\prime }:=S_{t}^{C}\wedge S_{t}^{*}$ and ${\bar{S}}_{t}^{\prime }:=S_{t}^{C}\vee S_{t}^{*}$. Then (8) implies that ${\bar{S}}_{t}^{\prime }-{\underline{S}}_{t}^{\prime }\leq B\left(t\right)\varepsilon$. Therefore, the model consisting of ${\underline{S}}_{t}^{\prime },{\bar{S}}_{t}^{\prime },S_{t}^{C},S_{t}^{*}$ is ε‐consistent. Conversely, assume that the given prices are ε‐consistent. Then there exist processes $S^{C}$ and $S^{*}$ on a probability space (Ω,F,P) such that $|S_{t}^{C}-S_{t}^{*}|\leq B\left(t\right)\varepsilon$ a.s. We then simply set ${\underline{S}}_{t}=S_{t}^{C}-B\left(t\right)\varepsilon$ and ${\bar{S}}_{t}=S_{t}^{C}+B\left(t\right)\varepsilon$, and have thus constructed an arithmetically 2ε‐consistent model.□

Note that the statement of Proposition 2.5 does not hold for consistency (instead of ε‐consistency), nor does it hold if we replace (8) with

$$\begin{array}{c} S_{t}^{C}=p{\underline{S}}_{t}+\left(1-p\right){\bar{S}}_{t},\;t\in T^{*}, \end{array}$$

where p∈[0,1] and $p\neq \frac{1}{2}$.

The process ${\left(D\left(t\right)S_{t}^{C}\right)}_{t\in T}$ does not have to be a martingale, as $S^{C}$ is not traded on the market. The option prices give us some information about the marginals of the process $S^{C}$, though. On the other hand, the process ${\left(D\left(t\right)S_{t}^{*}\right)}_{t\in T}$ has to be a martingale, but we have no information about its marginals, except that $|S_{t}^{*}-S_{t}^{C}|\leq \varepsilon B\left(t\right)$. This implies

(9) $$W^{\infty }\left(L\left(D\left(t\right)S_{t}^{C}\right),L\left(D\left(t\right)S_{t}^{*}\right)\right)\leq \varepsilon ,\;t\in T^{*},$$

where $W^{\infty }$ denotes the infinity Wasserstein distance, and L the law of a random variable. The distance $W^{\infty }$ is defined on M, the set of probability measures on R with finite mean, by

$$W^{\infty }\left(\mu ,\nu \right)=\inf{\left\|X-Y\right\|}_{\infty },\;\mu ,\nu \in M.$$

The infimum is taken over all probability spaces (Ω,F,P) and random pairs (X,Y) with marginals (μ,ν). See Gerhold and Gülüm (2019) for some references on $W^{\infty }$. For ε≥0 and random variables *X* and *Y*, the condition $W^{\infty }\left(LX,LY\right)\leq \varepsilon$ is equivalent to the existence of a probability space with random variables $X^{\prime }∼LX$, $Y^{\prime }∼LY$ such that $|X^{\prime }-Y^{\prime }|\leq \varepsilon$ a.s. (This is another result due to Strassen, see Proposition 4.6.)

Definition 2.6 Let μ,ν be two measures in M. Then we say that μ is smaller in *convex order* than ν, in symbols $\mu \leq_{c}\nu$, if for every convex function φ:R→R we have that ∫φdμ≤∫φdν, as long as both integrals are well defined. A family of measures ${\left(\mu_{t}\right)}_{t\in T^{*}}$ in M is called a *peacock*, if $\mu_{s}\leq_{c}\mu_{t}$ for all s≤t in $T^{*}$ (see Definition 1.3 in Hirsch, Profeta, Roynette, & Yor, 2011).

For μ∈M and x∈R, we define

(10) $$R_{\mu }\left(x\right)=\int_{R}{\left(y-x\right)}^{+}\mu \left(\mathrm{dy}\right),$$

the call function of μ. The mean of a measure μ will be denoted by Eμ=∫yμ(dy). These notions are useful for constructing models for ε‐consistent prices, as made explicit by the following lemma. As is evident from its proof, the sequence $\left(\mu_{t}\right)$ consists of the marginals of a (discounted) reference price, whereas $\left(\nu_{t}\right)$ gives the marginals of a martingale within the bid–ask spread. The proof uses a coupling result from our companion paper (Lemma 9.1 in Gerhold & Gülüm, 2019).

Lemma 2.7 For ε≥0, the prices (1)–(4) are ε‐consistent with the absence of arbitrage, if and only if ${\bar{S}}_{0}-{\underline{S}}_{0}\leq \varepsilon$ and there are sequences of finitely supported measures ${\left(\mu_{t}\right)}_{t\in T^{*}}$ and ${\left(\nu_{t}\right)}_{t\in T^{*}}$ in M such that:

(i) $R_{\mu_{t}}\left(k_{t,i}\right)\in \left[{\underline{r}}_{t,i},{\bar{r}}_{t,i}\right]$ for all $t\in T^{*}$ and $i\in \{1,\cdots ,N_{t}\}$, and $\mu_{t}\left(\left[\varepsilon ,\infty \right)\right)=1$ for $t\in T^{*}$,
(ii) ${\left(\nu_{t}\right)}_{t\in T^{*}}$ is a peacock and its mean satisfies $E\nu_{T}\in \left[{\underline{S}}_{0},{\bar{S}}_{0}\right]$, and
(iii) $W^{\infty }\left(\mu_{t},\nu_{t}\right)\leq \varepsilon$ for all $t\in T^{*}$.

Let ${\left(\mu_{t}\right)}_{t\in T^{*}}$ and ${\left(\nu_{t}\right)}_{t\in T^{*}}$ be as above. Recall that Strassen's (1965) Theorem 8 asserts that any peacock is the sequence of marginals of a martingale. Therefore, there is a finite filtered probability space with a martingale ${\left({\tilde{S}}_{t}\right)}_{t\in T}$ such that $\nu_{t}$ is the law of ${\tilde{S}}_{t}$ for $t\in T^{*}$.

From (iii), and the remark before Definition 2.6, it follows that there is a probability space with processes $\hat{M}$ and ${\hat{S}}^{C}$ such that ${\hat{M}}_{t}∼\nu_{t}$,$D\left(t\right){\hat{S}}_{t}^{C}∼\mu_{t}$, and $|{\hat{M}}_{t}-D\left(t\right){\hat{S}}_{t}^{C}|\leq \varepsilon$ for $t\in T^{*}$. As in the the proof of Theorem 9.2 in Gerhold and Gülüm (2019), it is easy to see that the finite support condition implies that there is a *finite* probability space with these properties. The sufficiency statement now easily follows from Lemma 9.1 in Gerhold and Gülüm (2019). Indeed, that lemma yields a finite filtered probability space with adapted processes ${\left({\overset{ˇ}{S}}_{t}\right)}_{t\in T}$ and ${\left(S_{t}^{C}\right)}_{t\in T^{*}}$ satisfying

- $\overset{ˇ}{S}$ is a martingale,
- ${\overset{ˇ}{S}}_{t}∼\nu_{t}$ and $D\left(t\right)S_{t}^{C}∼\mu_{t}$ for $t\in T^{*}$,
- $|{\overset{ˇ}{S}}_{t}-D\left(t\right)S_{t}^{C}|\leq \varepsilon$ for $t\in T^{*}$.

It then suffices to define

$$S_{t}^{*}:=B\left(t\right){\overset{ˇ}{S}}_{t},\;{\underline{S}}_{t}:=S_{t}^{C}\wedge S_{t}^{*},\;{\bar{S}}_{t}:=S_{t}^{C}\vee S_{t}^{*},\;t\in T^{*},$$

to obtain an arbitrage‐free model. Note that the second assertion in (ii) ensures that ${\underline{S}}_{t}\leq S_{t}^{*}\leq {\bar{S}}_{t}$ holds for t∈T and not just $T^{*}$.

Conversely, assume now that the given prices are ε‐consistent. For $t\in T^{*}$, define $\mu_{t}$ as the law of $D\left(t\right)S_{t}^{C}$, and $\nu_{t}$ as the law of $S_{t}^{*}$. It is then very easy to see that the stated conditions are satisfied. As for the finite support condition, note that the probability space in Definition 2.1 is finite.□

To prepare for the central notions of model‐independent and weak arbitrage, we now define semistatic trading strategies in the bank account, the underlying asset, and the call options. Here, semistatic means that the position in the call options is fixed at time zero. The definition is model‐independent; as soon as a model (in the sense of Definition 2.1) is chosen, the number of risky shares in the *t*th trading period, for example, becomes

(11) $$\varphi_{t}^{1}\left({\left({\underline{S}}_{u}\right)}_{1\leq u<t},{\left(S_{u}^{C}\right)}_{1\leq u<t},{\left({\bar{S}}_{u}\right)}_{1\leq u<t}\right),\;t\in T^{*}.$$

Definition 2.8

(i) A *semistatic portfolio*, or *semistatic trading strategy*, is a triple
  $$\Phi =\left({\left(\varphi_{t}^{0}\right)}_{t\in T^{*}},{\left(\varphi_{t}^{1}\right)}_{t\in T^{*}},{\left(\varphi^{t,i}\right)}_{t\in T^{*},\;i\in \left\{1,\cdots ,N_{t}\right\}}\right),$$
  where $\varphi_{1}^{0}\in R$, $\varphi_{t}^{0}:{\left(0,\infty \right)}^{3t}\to R$ are Borel measurable for $t\in T^{*}$, analogously for ϕ^1^, and $\varphi^{t,i}\in R$ for $t\in T^{*},i\in \left\{1,\cdots ,N_{t}\right\}$. Here, $\varphi_{t}^{0}$ denotes the investment in the bank account, $\varphi_{t}^{1}$ denotes the number of stocks held in the period from t−1 to *t*, and $\varphi^{t,i}\in R$ is the number of options with maturity $t\in T^{*}$ and strike $K_{t,i}$ which the investor buys at time zero.
(ii) A semistatic portfolio is called *self‐financing*, if
  $$\begin{array}{ccc} \varphi_{t+1}^{0}\left(s_{t}\right) & = & \frac{B(t+1)}{B(t)}\varphi_{t}^{0}\left(s_{t-1}\right)+\sum_{i=1}^{N_{t}}\varphi^{t,i}{\left(s_{t}^{C}-K_{t,i}\right)}^{+}\\ & & -\;{\left(\varphi_{t+1}^{1}\left(s_{t}\right)-\varphi_{t}^{1}\left(s_{t-1}\right)\right)}^{+}{\bar{s}}_{t}+{\left(\varphi_{t+1}^{1}\left(s_{t}\right)-\varphi_{t}^{1}\left(s_{t-1}\right)\right)}^{-}{\underline{s}}_{t} \end{array}$$
  holds for 1≤t<T and ${\underline{s}}_{u},s_{u}^{C},{\bar{s}}_{u}\in \left(0,\infty \right)$, 1≤u≤t, where
  (12) $$s_{t}:=\left({\left({\underline{s}}_{u}\right)}_{1\leq u\leq t},{\left(s_{u}^{C}\right)}_{1\leq u\leq t},{\left({\bar{s}}_{u}\right)}_{1\leq u\leq t}\right).$$
(iii) For prices (1)–(4), the *initial portfolio value* of a semistatic portfolio Φ is given by
  $$r_{\Phi }:=\varphi_{1}^{0}+{\left(\varphi_{1}^{1}\right)}^{+}{\bar{S}}_{0}-{\left(\varphi_{1}^{1}\right)}^{-}{\underline{S}}_{0}+\sum_{t\in T^{*}}\sum_{i=1}^{N_{t}}\left({\left(\varphi^{t,i}\right)}^{+}{\bar{r}}_{t,i}-{\left(\varphi^{t,i}\right)}^{-}{\underline{r}}_{t,i}\right).$$
  This is the cost of setting up the portfolio Φ.
(iv) The *liquidation value* at time *T* is defined as
  $$L_{\Phi }\left(s_{T}\right):=\frac{B(T)}{B(T-1)}\varphi_{T}^{0}\left(s_{T-1}\right)+\sum_{i=1}^{N_{T}}\varphi^{T,i}{\left(s_{T}^{C}-K_{T,i}\right)}^{+}-{\left(\varphi_{T}^{1}\left(s_{T-1}\right)\right)}^{-}{\bar{s}}_{T}+{\left(\varphi_{T}^{1}\left(s_{T-1}\right)\right)}^{+}{\underline{s}}_{T}.$$

Having defined semistatic portfolios, we can now formulate two useful notions of arbitrage.

Definition 2.9 Let ε≥0. The prices (1)–(4) admit *model‐independent arbitrage with respect to spread‐bound *ε, if we can form a self‐financing semistatic portfolio Φ in the bank account, the underlying asset and the options, such that the initial portfolio value $r_{\Phi }$ is negative and the following holds: For all real numbers ${\underline{s}}_{t},s_{t}^{C},{\bar{s}}_{t}\in \left(0,\infty \right)$, 1≤t≤T, that satisfy

$$\begin{array}{cc} 0<{\underline{s}}_{t}\leq s_{t}^{C}\leq {\bar{s}}_{t}, & \;t\in T^{*},\\ {\bar{s}}_{t}-{\underline{s}}_{t}\leq \varepsilon B\left(t\right), & \;t\in T^{*},\\ s_{t}^{C}\geq \varepsilon B\left(t\right), & \;t\in T^{*}, \end{array}$$

(cf. (5), (6), and (7)), we have $L_{\Phi }\left(s_{T}\right)\geq 0$.

Definition 2.10 Let ε≥0. The prices (1)–(4) admit a *weak arbitrage opportunity with respect to spread‐bound *ε if there is no model‐independent arbitrage strategy (with respect to spread‐bound ε), but for any model satisfying (6) and (7), there is a semistatic portfolio Φ such that the initial portfolio value $r_{\Phi }$ is nonpositive,

$$L_{\Phi }\left({\left({\underline{S}}_{u}\right)}_{1\leq u\leq T},{\left(S_{u}^{C}\right)}_{1\leq u\leq T},{\left({\bar{S}}_{u}\right)}_{1\leq u\leq T}\right)\geq 0,$$

and

$$P\left(L_{\Phi }\left({\left({\underline{S}}_{u}\right)}_{1\leq u\leq T},{\left(S_{u}^{C}\right)}_{1\leq u\leq T},{\left({\bar{S}}_{u}\right)}_{1\leq u\leq T}\right)>0\right)>0.$$

Most of the time we will fix ε≥0 and write only *model‐independent arbitrage*, meaning model‐independent arbitrage with respect to spread‐bound ε, and similarly for weak arbitrage. The notion of weak (i.e., model‐dependent) arbitrage was first used in Davis and Hobson (2007), where the authors give examples to highlight the distinction between weak arbitrage and model‐independent arbitrage. The crucial difference is that a weak arbitrage opportunity may depend on the null sets of the model. For example, suppose that we would like to use two different arbitrage strategies according to whether a certain call will expire in the money with positive probability or not. Such portfolios could serve to exhibit weak arbitrage (Definition 2.10), but will not show model‐independent arbitrage (Definition 2.9).

## 3 SINGLE MATURITY: ε‐CONSISTENCY

In this section, we characterize ε‐consistency (according to Definition 2.4) in the special case that all option maturities agree. The consistency conditions for a single maturity are similar to those derived in Theorem 3.1 of Davis and Hobson (2007) and Proposition 3 of Cousot (2007). In addition to the conditions given there, we have to assume that the mean of $S_{1}^{C}$ is “close enough” to *S*_0_.

We fix t=1∈T and often drop the time index for notational convenience, that is, we write ${\bar{r}}_{i}$ instead of ${\bar{r}}_{1,i}$. In the frictionless case, the underlying can be identified with an option with strike k=0. Here, we will do something similar: in the formulation of the next theorem we set $k_{0}=\varepsilon$, as if we would introduce an option with strike εB(1), but we think of C(εB(1)) as the underlying. The choices for ${\underline{r}}_{0}={\underline{S}}_{0}-2\varepsilon$ and ${\bar{r}}_{0}={\bar{S}}_{0}$ made in Theorem 3.1 can be motivated as follows: in every model that is ε‐consistent with the absence of arbitrage, (7) implies that the discounted expected payoff of an option with strike εB(1) has to satisfy

$$D\left(1\right)E\left[{\left(S_{1}^{C}-\varepsilon B\left(1\right)\right)}^{+}\right]=D\left(1\right)E\left[S_{1}^{C}\right]-\varepsilon .$$

Furthermore, to guarantee the existence of a consistent price system, $D\left(1\right)E\left[S_{1}^{C}\right]$ has to lie in the closed interval $\left[{\underline{S}}_{0}-\varepsilon ,{\bar{S}}_{0}+\varepsilon \right]$, which implies that the price of an option with strike B(1)ε has to lie in the interval $\left[{\underline{S}}_{0}-2\varepsilon ,{\bar{S}}_{0}\right]$. Therefore, in the proof of Theorem 3.1 (given in Appendix A), we will use the symbol $C_{t}\left(\varepsilon B\left(t\right)\right)$ as a reference to the underlying and $-C_{t}\left(\varepsilon B\left(t\right)\right)$ as a reference to a short position in the underlying plus an additional deposit of 2ε in the bank account.

Before we formulate the main result for a single maturity, we recall that a butterfly contract (with maturity 1) is defined by

$$\frac{1}{K_{j}-K_{i}}C_{1}\left(K_{i}\right)-\left(\frac{1}{K_{j}-K_{i}}+\frac{1}{K_{l}-K_{j}}\right)C_{1}\left(K_{j}\right)+\frac{1}{K_{l}-K_{j}}C_{1}\left(K_{l}\right),$$

where 0≤i<j<l≤N, and that its payoff is nonnegative. A call spread is a portfolio of a long and a short call, where the latter has a larger strike.

Theorem 3.1 Let ε≥0 and consider prices as at the beginning of Section 2, with T=1 and $k_{1}>\varepsilon$ (see the remarks after (7)). Moreover, for ease of notation (see the above remarks) we set $k_{0}=\varepsilon$, ${\underline{r}}_{0}={\underline{S}}_{0}-2\varepsilon$, and ${\bar{r}}_{0}={\bar{S}}_{0}$. Then the prices are ε‐consistent (see Definition 2.4) if and only if the following conditions hold:

(i) All butterfly spreads have nonnegative time‐0 price, that is,
  (13) $$\frac{{\bar{r}}_{l}-{\underline{r}}_{j}}{k_{l}-k_{j}}\geq \frac{{\underline{r}}_{j}-{\bar{r}}_{i}}{k_{j}-k_{i}},\;0\leq i<j<l\leq N.$$
(ii) The call prices satisfy
  (14) $$\frac{{\bar{r}}_{l}-{\underline{r}}_{i}}{k_{l}-k_{i}}\geq -1,\;0\leq i<l\leq N.$$
(iii) All call spreads have nonnegative time‐0 price, that is,
  (15) $${\underline{r}}_{j}\leq {\bar{r}}_{i},\;0\leq i<j\leq N.$$
(iv) If a call spread is available for zero cost, then the involved options have zero bid, respectively, ask price, that is,
  (16) $${\underline{r}}_{j}={\bar{r}}_{i}\;\Rightarrow \;{\underline{r}}_{j}={\bar{r}}_{i}=0,\;0\leq i<j\leq N.$$

Moreover, there is a model‐independent arbitrage, as soon as any of the conditions (i)–(iii) is not satisfied. Finally, if (i)–(iii) hold but (iv) fails, then there is a weak arbitrage opportunity.

This theorem is proved in Appendices A and B. We conclude that the trichotomy of consistency/weak arbitrage/model‐independent arbitrage, which was uncovered by Davis and Hobson (2007) in the frictionless case, persists under bid–ask spreads (at least in the one‐period setting).

For ε=0 and ${\underline{r}}_{i}={\bar{r}}_{i}=r_{i}$, the conditions from Theorem 3.1 simplify to

$$0\geq \frac{r_{i+1}-r_{i}}{k_{i+1}-k_{i}}\geq \frac{r_{i}-r_{i-1}}{k_{i}-k_{i-1}}\geq -1,\;\text{for}\;i\in \left\{1,\cdots ,N-1\right\},$$

and

$$r_{i}=r_{i-1}\;\text{implies}\;r_{i}=0,\;\text{for}\;i\in \left\{1,\cdots ,N\right\}.$$

These are exactly the conditions required in Theorem 3.1 of Davis and Hobson (2007).

Remark 3.2 Note that in contrast to the frictionless case, it is not required that bid or ask prices decrease as the strike increases, in order to get models that are ε‐consistent with the absence of arbitrage. This means that we do not have to require ${\underline{r}}_{i}\geq {\underline{r}}_{j}$ or ${\bar{r}}_{i}\geq {\bar{r}}_{j}$ for i<j, as shown in the following example.

**Figure 1:**
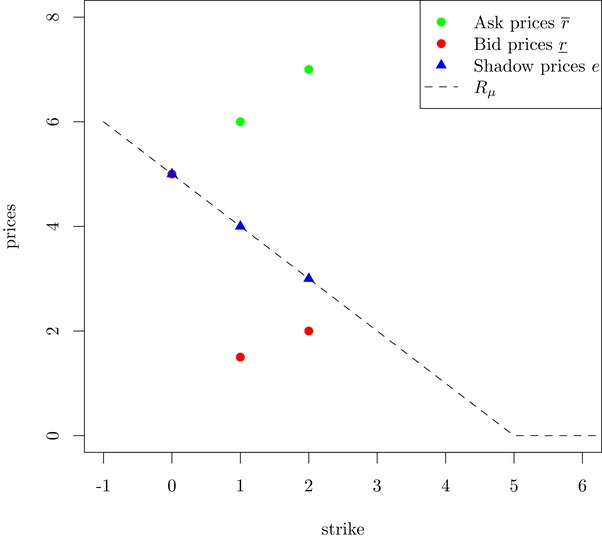
This example shows that it is not necessary that the ask‐prices, respectively, bid‐prices decrease with respect to strike. The line represents the call function of δ_5_ [Color figure can be viewed at http://wileyonlinelibrary.com]

Consider two call options, where ε=0 (no spread on the underlying), and the prices are given by ${\underline{S}}_{0}={\bar{S}}_{0}=5,\;{\bar{r}}_{i}=i+5,\;{\underline{r}}_{i}=1+\frac{i}{2},k_{i}=i$ for i=1,2. We assume that the bank account is constant until maturity. These prices and a possible choice of shadow prices $e_{i}:=D\left(1\right)E\left[{\left(S_{1}^{C}-K_{i}\right)}^{+}\right]$ are shown in Figure 1. (Note that shadow prices are introduced in the proof of Theorem 3.1 in Appendix A.) Clearly, all conditions from Theorem 3.1 are satisfied, and therefore there exists an arbitrage‐free model. For example, we can choose $\mu =\delta_{5}$, where δ denotes the Dirac delta. This example shows that, in our setting, prices that are admissible from a no‐arbitrage point of view do not necessarily make economic sense: As the payoff of $C(K_{2})$ at maturity never exceeds the payoff of $C(K_{1})$, the utility indifference ask‐price of $C(K_{2})$ should not be higher than the utility indifference ask‐price of $C(K_{1})$.

From Theorem 3.1, it is easy to explicitly compute the interval of all ε such that the given prices are ε‐consistent, which completes the solution of the ε‐consistency problem in the one‐period case. Note that (13)–(16) clearly have to be satisfied for i,j,l>0, as these conditions depend on ε only for i=0 (see also Proposition 4.1).

Corollary 3.3 Assume that the given prices satisfy Equations (13)–(16) for i,j,l>0. Then for ε≥0, the prices are ε‐consistent with the absence of arbitrage if and only if ε satisfies:

$$\begin{array}{cc} \varepsilon & \geq \max\left\{{\bar{S}}_{0}-{\underline{S}}_{0},{\underline{S}}_{0}-\left({\bar{r}}_{i}-k_{i}\right),k_{j}-\frac{{\underline{r}}_{j}-{\bar{S}}_{0}}{{\bar{r}}_{l}-{\underline{r}}_{j}}\cdot \left(k_{l}-k_{j}\right)\right\},\\ & \;\;\;1\leq i\leq N,\;1\leq j<l\leq N\;\text{such}\;\text{that}\;{\bar{r}}_{l}>{\underline{r}}_{j},\\ \varepsilon & \leq \min\left\{k_{1},k_{j}-\frac{{\underline{r}}_{j}-{\bar{S}}_{0}}{{\bar{r}}_{l}-{\underline{r}}_{j}}\cdot \left(k_{l}-k_{j}\right)\right\},\;1\leq j<l\leq N\;\text{such}\;\text{that}\;{\bar{r}}_{l}<{\underline{r}}_{j}. \end{array}$$

First, the inequalities $\varepsilon \geq {\bar{S}}_{0}-{\underline{S}}_{0}$ and $\varepsilon \leq k_{1}$ follow from the definition of ε‐consistency (see (6) and (7)). The remaining inequalities follow by setting i=0 in (13) and (14).□

## 4 MULTIPLE MATURITIES: EQUIVALENT CONDITIONS FOR CONSISTENCY AND ε‐CONSISTENCY

As mentioned in Section 1, our main goal is to find the least bound on the underlying's bid–ask spread that enables us to reproduce given option prices. The following result clarifies the situation if *no* such bound is imposed (see also Example 2.3). In our wording, we first seek conditions for consistency (Definition 2.2) and not ε‐consistency (Definition 2.4). Recall the notation used in, and explained before, Theorem 3.1, where i=0 is allowed in (13)–(16), inducing a dependence of these conditions on ${\underline{S}}_{0}$ and ${\bar{S}}_{0}$. In the following proposition, on the other hand, we require i,j,l≥1, and therefore the current bid and ask prices of the underlying are irrelevant when checking consistency of option prices. Thus, the notion of ε‐consistency seems to make more sense than consistency.

Proposition 4.1 The prices (1)–(4) are consistent with the absence of arbitrage (see Definition 2.2) if and only if, for all $t\in T^{*}$, the conditions (13)–(16) from Theorem 3.1 hold for $i,j,l\in \{1,\cdots ,N_{t}\}$.

By mimicking the proof of the first part of Theorem 3.1 for i,j,l>0, we see that the conditions are necessary. Now fix $t\in T^{*}$ and assume that the conditions hold. Exactly as in the sufficiency proof of Theorem 3.1, we can construct $e_{t,1},e_{t,2},\cdots ,e_{t,N_{t}}$ such that $e_{t,i}\in \left[{\underline{r}}_{t,i},{\bar{r}}_{t,i}\right]$. The linear interpolation $L_{t}$ of the points ${\left(k_{t,i},e_{t,i}\right)}_{i\in \{1,\cdots ,N_{t}\}}$ can then be extended to a call function of a measure $\mu_{t}$ (see the final part of the sufficiency proof of Theorem 3.1).

We define random variables $S_{t}^{C}$ such that the law of $D\left(t\right)S_{t}^{C}$ is given by $\mu_{t}$. Then we have that

$$D\left(t\right)E\left[{\left(S_{t}^{C}-K_{t,i}\right)}^{+}\right]=e_{t,i}\in \left[{\underline{r}}_{t,i},{\bar{r}}_{t,i}\right],\;i\in \left\{1,\cdots N_{t}\right\}.$$

Furthermore, we pick $s\in [{\underline{S}}_{0},{\bar{S}}_{0}]$ and set $\nu_{t}=\delta_{s}$ (Dirac delta) for all $t\in T^{*}$. Clearly, ${\left(\nu_{t}\right)}_{t\in T^{*}}$ is a peacock, and we set $S_{t}^{*}=B\left(t\right)s$, which implies $D\left(t\right)S_{t}^{*}∼\nu_{t}$. Finally, we define ${\underline{S}}_{t}=S_{t}^{*}\wedge S_{t}^{C}$ and ${\bar{S}}_{t}=S_{t}^{*}\vee S_{t}^{C}$, and have thus constructed an arbitrage‐free model.□

To prepare for our main result on ε‐consistency in the multiperiod model, we now recall the main result of Gerhold and Gülüm (2019), which gives a criterion for the existence of the peacock $\left(\nu_{t}\right)$ from Lemma 2.7. Recall also the notation $W^{\infty },M$ introduced before Definition 2.6. According to Proposition 3.2 in Gerhold and Gülüm (2019), for ε>0, a measure μ∈M, and m∈[Eμ−ε,Eμ+ε], the set

$$\left\{\nu \in M:W^{\infty }\left(\mu ,\nu \right)\leq \varepsilon ,\;E\nu =m\right\}$$

has a smallest and a largest element, and their respective call functions can be expressed explicitly by the call function $R_{\mu }$ of μ (see (10)) as follows:

$$\begin{array}{cc} R_{\mu }^{\min}\left(x;m,\varepsilon \right) & =\left(m+R_{\mu }\left(x-\varepsilon \right)-\left(E\mu +\varepsilon \right)\right)\vee R_{\mu }\left(x+\varepsilon \right),\\ R_{\mu }^{\max}\left(x;m,\varepsilon \right) & =\mathrm{conv}\left(m+R_{\mu }\left(\cdot +\varepsilon \right)-\left(E\mu -\varepsilon \right),R_{\mu }\left(\cdot -\varepsilon \right)\right)\left(x\right), \end{array}$$

where conv denotes the convex hull. The main theorem of Gerhold and Gülüm (2019) gives an equivalent condition for the existence of a peacock within $W^{\infty }$‐distance ε of a given sequence of measures.

Theorem 4.2. (Theorem 3.5 in Gerhold and Gülüm (2019)) Let ε>0 and ${\left(\mu_{n}\right)}_{n\in N}$ be a sequence in M such that

$$I:=\cap_{n\in N}\left[E\mu_{n}-\varepsilon ,E\mu_{n}+\varepsilon \right]$$

is not empty. Then there exists a peacock ${\left(\nu_{n}\right)}_{n\in N}$ such that

(17) $$W^{\infty }\left(\mu_{n},\nu_{n}\right)\leq \varepsilon ,\;\text{for}\;\text{all}\;n\in N,$$

if and only if for some m∈I and for all N∈N, $x_{1},\cdots ,x_{N}\in R$, we have

(18) $$\begin{array}{c} R_{\mu_{1}}^{\min}\left(x_{1};m,\varepsilon \right)+\sum_{n=2}^{N}\left(R_{\mu_{n}}\left(x_{n}+\varepsilon \sigma_{n}\right)-R_{\mu_{n}}\left(x_{n-1}+\varepsilon \sigma_{n}\right)\right)\leq R_{\mu_{N+1}}^{\max}\left(x_{N};m,\varepsilon \right). \end{array}$$

Here, $\sigma_{n}=\mathrm{sgn}\left(x_{n-1}-x_{n}\right)$ depends on $x_{n-1}$ and $x_{n}$. In this case, it is possible to choose $E\nu_{1}=E\nu_{2}=\cdots =m$.

We can now give a partial solution to the multiperiod ε‐consistency problem. The existence of the measures $\mu_{t}$ from Lemma 2.7 (the marginals of $DS^{C}$) has to be assumed, but the existence of the peacock $\left(\nu_{t}\right)$ can be replaced by fairly explicit conditions, using Theorem 4.2.

Theorem 4.3 For ε≥0 the prices (1)–(4) are ε‐consistent with the absence of arbitrage, if and only if ${\bar{S}}_{0}-{\underline{S}}_{0}\leq \varepsilon$ and there is a sequence of finitely supported measures ${\left(\mu_{t}\right)}_{t\in T^{*}}$ in M such that:

(i) $R_{\mu_{t}}\left(k_{t,i}\right)\in \left[{\underline{r}}_{t,i},{\bar{r}}_{t,i}\right]$ for all $t\in T^{*}$ and $i\in \{1,\cdots ,N_{t}\}$, and $\mu_{t}\left(\left[\varepsilon ,\infty \right)\right)=1$ for $t\in T^{*}$,
(ii) There is
  $$m\in \cap_{t\in T^{*}}\left[E\mu_{t}-\varepsilon ,E\mu_{t}+\varepsilon \right]\cap \left[{\underline{S}}_{0},{\bar{S}}_{0}\right]$$
  such that for all N∈{1,⋯,T−1} and $x_{1},\cdots ,x_{N}\in R$
  $$R_{\mu_{1}}^{\min}\left(x_{1};m,\varepsilon \right)+\sum_{n=2}^{N}\left(R_{\mu_{n}}\left(x_{n}+\varepsilon \sigma_{n}\right)-R_{\mu_{n}}\left(x_{n-1}+\varepsilon \sigma_{n}\right)\right)\leq R_{\mu_{N+1}}^{\max}\left(x_{N};m,\varepsilon \right),$$
  where $\sigma_{n}$ is as in Theorem 4.2. and $\mu_{n}:=\mu_{T}$ for n>T.

Immediate from Lemma 2.7 and Theorem 4.2. □

As we allow an arbitrary reference price process $S^{C}$ in Definitions 2.1 and 2.2, our notion of consistency is fairly weak. It can be weakened further by requiring that the bound (6) holds only with a certain probability instead of almost surely. However, according to the following theorem, we can always find such a model as soon as the prices are consistent.

Theorem 4.4 Let p∈(0,1] and ε≥0. For given prices (1)–(4), the following are equivalent:

(i) The prices satisfy Definition 2.4 (ε‐consistency), but with (6) replaced by the weaker condition
  $$P\left({\bar{S}}_{t}-{\underline{S}}_{t}\geq \varepsilon B\left(t\right)\right)\leq p,\;t\in T.$$
(ii) The prices are consistent with the absence of arbitrage.

For the proof of Theorem 4.4, we employ a result from Gerhold and Gülüm (2019) on the modified Prokhorov distance.

Definition 4.5 For p∈[0,1] and two probability measures μ,ν on R, we define the *modified Prokhorov distance* as

$$\begin{array}{c} d_{p}^{P}\left(\mu ,\nu \right):=\inf\left\{h>0:\nu \left(A\right)\leq \mu \left(A^{h}\right)+p,\;\text{for}\;\text{all}\;\text{closed}\;\text{sets}\;A\subseteq R\right\}. \end{array}$$

(To define the standard Prokhorov distance, replace *p* by *h* in the right‐hand side.) Note that $d_{0}^{P}=W^{\infty }$. A well‐known result, which was first proved by Strassen (1965), and was then extended by Dudley (1968), explains the connection of $d_{p}^{P}$ to minimal distance couplings.

Proposition 4.6 Given measures μ,ν on R, p∈[0,1], and ε>0, there exists a probability space (Ω,F,P) with random variables X∼μ and Y∼ν such that

(19) P(|X−Y|>ε)≤p,

if and only if

(20) $$d_{p}^{P}\left(\mu ,\nu \right)\leq \varepsilon .$$

The following result shows that, unlike for $W^{\infty }$, there *always* exists an approximating peacock with respect to $d_{p}^{P}$ for 0<p≤1. This explains why the very weak condition of consistency is sufficient to imply (i) in Theorem 4.4.

Theorem 4.7. (Theorem 8.3 in Gerhold and Gülüm (2019)) Let ${\left(\mu_{n}\right)}_{n\in N}$ be a sequence in M, ε>0, and p∈(0,1]. Then, for all m∈R there exists a peacock ${\left(\nu_{n}\right)}_{n\in N}$ with mean *m* such that

$$d_{p}^{P}\left(\mu_{n},\nu_{n}\right)\leq \varepsilon .$$

Proof of Theorem 4.4 (i) implies (ii) by definition. To show the other implication, we define probability measures ${\left(\mu_{t}\right)}_{t\in T^{*}}$ as in the proof of Proposition 4.1, such that $R_{\mu_{t}}\left(k_{t,i}\right)\in \left[{\underline{r}}_{t,i},{\bar{r}}_{t,i}\right]$ for $i\in \{1,\cdots N_{t}\}$ and $t\in T^{*}$. Now we pick $s\in [{\underline{S}}_{0},{\bar{S}}_{0}]$. Then by, Theorem 4.7., there exists a peacock ${\left(\nu_{t}\right)}_{t\in T^{*}}$ with mean *s* such that $d_{p}^{P}\left(\mu_{t},\nu_{t}\right)\leq \varepsilon$ for all $t\in T^{*}$. We can now use Proposition 4.6 and proceed as in the proof of Lemma 2.7 to conclude that there exist stochastic processes ${\left({\tilde{S}}_{t}^{C}\right)}_{t\in T^{*}}$ and ${\left({\tilde{S}}_{t}^{*}\right)}_{t\in T^{*}}$ whose marginal distributions are given by ${\left(\mu_{t}\right)}_{t\in T^{*}}$ , respectively, ${\left(\nu_{t}\right)}_{t\in T^{*}}$, such that ${\left({\tilde{S}}_{t}^{*}\right)}_{t\in T^{*}}$ is a martingale and such that

$$\begin{array}{c} P\left(\left|{\tilde{S}}_{t}^{*}-{\tilde{S}}_{t}^{C}\right|\geq \varepsilon \right)\leq p,\;t\in T^{*}. \end{array}$$

The coupling lemma we use (Lemma 9.1) was formulated in Gerhold and Gülüm (2019) for the special case p=0, but the proof trivially extends to p∈[0,1]. We then simply put

$$S_{t}^{*}=B\left(t\right){\tilde{S}}_{t}^{*},\;S_{t}^{C}=B\left(t\right){\tilde{S}}_{t}^{C},\;{\underline{S}}_{t}=S_{t}^{*}\wedge S_{t}^{C},\;\text{and}\;{\bar{S}}_{t}=S_{t}^{*}\vee S_{t}^{C}.$$

□

## 5 MULTIPLE MATURITIES: NECESSARY CONDITIONS FOR ε‐CONSISTENCY

The main result of the preceding section (Theorem 4.3) gives semiexplicit equivalent conditions for ε‐consistency. The goal of the present section is to provide explicit necessary conditions. For a single maturity, the ε‐consistency conditions (Theorem 3.1) are a generalization of the frictionless conditions in Cousot (2007) and Davis and Hobson (2007). They guarantee that for each maturity $t\in T^{*}$ the option prices can be associated to a measure $\mu_{t}$, such that $E\mu_{t}\in \left[{\underline{S}}_{0},{\bar{S}}_{0}\right]$ (cf. Lemma 2.7). In this section, we state *necessary* conditions for multiple periods. Our conditions (see Definition 5.1 and Theorem 5.3) are fairly involved, and we thus expect that it might not be easy to obtain tractable *equivalent* conditions. In the case where there is only a spread on the options, but not on the underlying, it suffices to compare prices with only three or two different maturities (see equations (4)– (6) in Cousot, 2007 and Corollary 4.2 in Davis & Hobson, 2007) to obtain suitable consistency conditions. These conditions ensure that the family of measures ${\left(\mu_{t}\right)}_{t\in T^{*}}$ is a peacock.

If we consider a bid–ask spread on the underlying and want to check for ε‐consistency according to Definition 2.4 (ε>0), it turns out that we need conditions that involve all maturities simultaneously (this will become clear by condition (18)). We thus introduce calendar vertical baskets (CVB), portfolios that consist of various long and short positions in the call options. We first give a definition of CVBs. Then, in Lemma 5.2 we will study a certain trading strategy involving a short position in a CVB. This strategy will then serve as a base for the conditions in Theorem 5.3, which is the main result of this section. Note that our definition of a CVB depends on ε≥0: the contract defined in Definition 5.1 only provides necessary conditions in markets where the bid–ask spread is bounded by ε≥0.

Definition 5.1 Fix u∈{1,⋯,T−1} and ε≥0 and assume that vectors $\sigma =(\sigma_{1},\cdots ,\sigma_{u})$, $x=(x_{1},\cdots ,x_{u})$, $I=(i_{2},\cdots ,i_{u})$ and $J=(j_{1},\cdots ,j_{u})$ are given, such that

(i) $x_{t}\in R$ for all t∈{1,⋯,u},
(ii) $\sigma_{1}\in \left\{-1,1\right\}$ and $\sigma_{t}=\mathrm{sgn}\left(x_{t-1}-x_{t}\right)$ for all t∈{2,⋯,u},
(iii) $j_{t}\in \left\{0,\cdots ,N_{t}\right\}$ and $k_{t,j_{t}}=x_{t}+\varepsilon \sigma_{t}$ for all t∈{1,⋯,u},
(iv) $i_{t}\in \left\{0,\cdots ,N_{t}\right\}$ and either $k_{t,i_{t}}\leq x_{t-1}+\varepsilon \sigma_{t}$ or $i_{t}=0$ for all t∈{2,⋯,u}.

Then, we define a CVB with these parameters as the contract

(21) $$\begin{array}{c} {\mathrm{CVB}}_{u}\left(\sigma ,x,I,J\right)=C_{1}\left(K_{1,j_{1}}\right)+\sum_{t=2}^{u}\left(C_{t}\left(K_{t,j_{t}}\right)-C_{t}\left(K_{t,i_{t}}\right)\right)-2\varepsilon 1_{\left\{\sigma_{1}=-1\right\}}. \end{array}$$

The market ask, respectively, bid‐price of $CVB_{u}\left(\sigma ,x,I,J\right)$ are given by

(22) $$\begin{array}{cc} {\bar{r}}_{u}^{\mathrm{CVB}}\left(\sigma ,x,I,J\right) & ={\bar{r}}_{1,j_{1}}+\sum_{t=2}^{u}\left({\bar{r}}_{t,j_{t}}-{\underline{r}}_{t,i_{t}}\right)-2\varepsilon 1_{\left\{\sigma_{1}=-1\right\}},\\ {\underline{r}}_{u}^{\mathrm{CVB}}\left(\sigma ,x,I,J\right) & ={\underline{r}}_{1,j_{1}}+\sum_{t=2}^{u}\left({\underline{r}}_{t,j_{t}}-{\bar{r}}_{t,i_{t}}\right)+2\varepsilon 1_{\left\{\sigma_{1}=-1\right\}}. \end{array}$$

We will refer to *u* as the maturity of the CVB.

Lemma 5.2 Fix ε≥0. For all parameters u,σ,x,I,J as in Definition 5.1, there is a self‐financing semistatic portfolio Φ whose initial value is given by $r_{\Phi }=-{\underline{r}}_{u}^{CVB}\left(\sigma ,x,I,J\right)$, such that for all models satisfying (6) and (7) and for all t∈{2,⋯,u+1} one of the following conditions holds:

(i) $\varphi_{t}^{0}\geq 0$ and $\varphi_{t}^{1}=0$, or
(ii) $\varphi_{t}^{0}\geq k_{t,j}-\varepsilon \sigma_{t}$ and $\varphi_{t}^{1}=-1$.

In particular, all corresponding cash flows are nonnegative.

The arguments of $\varphi_{t}^{0},\varphi_{t}^{1}$ are of course the same as in (11), and are omitted for brevity. In the proof of Lemma 5.2, we define the functions $\varphi_{t}^{0},\varphi_{t}^{1}$ inductively. As we are defining a model‐independent strategy, we could also use the deterministic dummy variables (12) from Definition 2.8 as arguments. It seems more natural to write ${\left({\underline{S}}_{u}\right)}_{u\leq t},{\left(S_{u}^{C}\right)}_{u\leq t},{\left({\bar{S}}_{u}\right)}_{u\leq t}$, though. We just have to keep in mind that $\varphi_{t}^{0},\varphi_{t}^{1}$ have to be constructed as *functions* of ${\left({\underline{S}}_{u}\right)}_{u\leq t},{\left(S_{u}^{C}\right)}_{u\leq t},{\left({\bar{S}}_{u}\right)}_{u\leq t}$, without using the *distribution* of these random vectors.

Moreover, note that later on in Theorem 5.3 we will only need the case where u<T, therefore we excluded the case u=T.

Proof of Lemma 5.2 Assume that we buy the contract

(23) $$\begin{array}{c} -CVB_{u}\left(\sigma ,x,I,J\right)=-C_{1}\left(K_{1,j_{1}}\right)+\sum_{t=2}^{u}\left(C_{t}\left(K_{t,i_{t}}\right)-C_{t}\left(K_{t,j_{t}}\right)\right)+2\varepsilon 1_{\left\{\sigma_{1}=-1\right\}}, \end{array}$$

thus we are getting an initial payment of ${\underline{r}}_{u}^{CVB}\left(\sigma ,x,I,J\right)$. We have to keep in mind that if $i_{t}=0$ for some t∈{2,⋯,u}, then the corresponding expression in (23) denotes a long position in the underlying, and if $j_{t}=0$ for some t∈{1,⋯,u}, then the expression $-C_{t}\left(K_{t,j_{t}}\right)$ in (23) denotes a short position in the underlying plus an additional deposit of 2ε in the bank account at time 0 (see the beginning of Section 3). To ease notation, we will write $K_{t,i}$ instead of $K_{t,i_{t}}$ and $K_{t,j}$ instead of $K_{t,j_{t}}$.

We will show inductively that after we have traded at time t∈{1,⋯,u} we can end up in one of two scenarios: either the investor holds a nonnegative amount of bank units (i.e., $\varphi_{t+1}^{0}\geq 0$), we will call this scenario A, or we have one short position in the underlying (i.e., $\varphi_{t+1}^{1}=-1$) and $\varphi_{t+1}^{0}\geq k_{t,j}-\varepsilon \sigma_{t}$; we will refer to this as scenario B. Note that scenarios A and B are not disjoint, but this will not be a problem.

We will first deal with the case where $\sigma_{1}=-1$ and afterward with the case $\sigma_{1}=1$. We start with t=1 and first assume that $j_{1}>0$. If $C_{1}\left(K_{1,j}\right)$ expires out of the money, then we do not trade at time 1 and obtain $\varphi_{2}^{0}=2\varepsilon \geq 0$, so we are in scenario A. Otherwise we sell one unit of the underlying, and thus

$$\varphi_{2}^{0}=2\varepsilon +k_{1,j}+D\left(1\right)\left({\underline{S}}_{1}-S_{1}^{C}\right)\geq k_{1,j}+\varepsilon =k_{1,j}-\sigma_{1}\varepsilon ,$$

yielding scenario B. Recall from Section 2 that $D\left(t\right)=B{\left(t\right)}^{-1}$. If $j_{1}=0$ then $k_{1,j}=\varepsilon$. We do not close the short position in this case and we get that $\varphi_{2}^{0}=4\varepsilon \geq k_{1,j}-\sigma_{1}\varepsilon$, so we also get to scenario B.

For the induction step, we split the proof into two parts. In part A, we will assume that after trading time t−1 we are in scenario A, and in part B, we will assume that at the end of period t−1 we are in scenario B.

Part A: We will show that after we have traded at time *t* we can end up either in situation A or B. First we assume that $j_{t},i_{t}>0$, and so both expressions in (23) with maturity *t* denote options (and not the underlying). Under these assumptions, $\varphi_{t}^{0}$ satisfies

$$\varphi_{t+1}^{0}\geq D\left(t\right){\left(S_{t}^{C}-K_{t,i}\right)}^{+}-D\left(t\right){\left(S_{t}^{C}-K_{t,j}\right)}^{+}.$$

Clearly, if $K_{t,i}\leq K_{t,j}$ or if both options expire out of the money, then $\varphi_{t+1}^{0}\geq 0$, and we are in situation A. So, suppose that $K_{t,i}>K_{t,j}$ and that $S_{t}^{C}>K_{t,j}$. This also implies that $\sigma_{t}=1$. If this is the case, we go short one unit of the underlying, and $\varphi_{t+1}^{0}$ can be bounded from below as follows:

$$\begin{array}{cc} \varphi_{t+1}^{0} & \geq D\left(t\right){\left(S_{t}^{C}-K_{t,i}\right)}^{+}-D\left(t\right)\left(S_{t}^{C}-K_{t,j}\right)+D\left(t\right){\underline{S}}_{t}\\ & \geq k_{t,j}-\varepsilon \sigma_{t}. \end{array}$$

This corresponds to situation B. Next assume that $j_{t}=0$ and $i_{t}>0$. Then we have that $k_{t,j}=\varepsilon$. After trading time *t*, we end up in scenario B,

$$\begin{array}{cc} \varphi_{t+1}^{0} & \geq D\left(t\right){\left(S_{t}^{C}-K_{t,i}\right)}^{+}+2\varepsilon \geq k_{t,j}-\varepsilon \sigma_{t}. \end{array}$$

We proceed with the case that $j_{t}>0$ and $i_{t}=0$. As $k_{t,j}>\varepsilon$, we can close the long position in the underlying and end up in scenario A at the end of time *t*,

$$\begin{array}{c} \varphi_{t+1}^{0}\geq D\left(t\right){\underline{S}}_{t}-D\left(t\right){\left(S_{t}^{C}-K_{t,j}\right)}^{+}\geq 0. \end{array}$$

The case where $j_{t}=i_{t}=0$ is easily handled, because the long and the short position simply cancel out. We are done with part A.

Part B: Assume that after we have traded at time t−1 we are in scenario B, and thus $\varphi_{t}^{0}=k_{t-1,j}-\varepsilon \sigma_{t-1}$. First we will consider the case where $j_{t},i_{t}>0$. If at time *t* the option with strike $K_{t,j}$ expires in the money, we do not close the short position and have

$$\begin{array}{cc} \varphi_{t+1}^{0} & \geq \varphi_{t}^{0}+D\left(t\right){\left(S_{t}^{C}-K_{t,i}\right)}^{+}-D\left(t\right)\left(S_{t}^{C}-K_{t,j}\right)\\ & =k_{t-1,j}-\varepsilon \sigma_{t-1}+k_{t,j}-k_{t,i}\\ & \geq k_{t,j}-\varepsilon \sigma_{t}, \end{array}$$

which means that we end up in scenario B. Now we distinguish two cases according to $x_{t-1}\leq x_{t}$ and $x_{t-1}>x_{t}$, and always assume that $C_{t}\left(K_{t,j}\right)$ expires out of the money. If $x_{t-1}\leq x_{t}$, then we also have that $k_{t,i}\leq k_{t,j}$ and that $\sigma_{t}=-1$. We close the short position to end up in scenario A,

$$\begin{array}{cc} \varphi_{t+1}^{0} & \geq \varphi_{t}^{0}+D\left(t\right){\left(S_{t}^{C}-K_{t,i}\right)}^{+}-D\left(t\right){\bar{S}}_{t}\\ & \geq k_{t,i}-\varepsilon \sigma_{t}-k_{t,i}-\varepsilon \geq 0. \end{array}$$

If, on the other hand, $x_{t-1}>x_{t}$ and $\sigma_{t}=1$, we do not trade at time *t* to stay in scenario B,

$$\begin{array}{ccc} \varphi_{t+1}^{0} & \geq & \varphi_{t}^{0}+D\left(t\right){\left(S_{t}^{C}-K_{t,i}\right)}^{+}\\ & > & k_{t,j}-\varepsilon \sigma_{t}. \end{array}$$

We proceed with the case where $j_{t}=0$ and $i_{t}>0$. As before, we have $k_{t,j}=\varepsilon$, and we can close one short position to stay in scenario B,

$$\begin{array}{cc} \varphi_{t+1}^{0} & =\varphi_{t}^{0}+D\left(t\right){\left(S_{t}^{C}-K_{t,i}\right)}^{+}+2\varepsilon -D\left(t\right){\bar{S}}_{t}\\ & \geq k_{t-1,j}-\varepsilon \sigma_{t-1}-k_{t,i}+\varepsilon \\ & \geq \varepsilon -\varepsilon \sigma_{t}=k_{t,j}-\varepsilon \sigma_{t}. \end{array}$$

If $j_{t}>0$ and $i_{t}=0$, then we distinguish two cases: either $C_{t}\left(K_{t,j}\right)$ expires out of the money, in which case we cancel out the long and short position in the underlying and have

$$\varphi_{t+1}^{0}\geq \varphi_{t}^{0}\geq 0,$$

which corresponds to scenario A. Or, $C_{t}\left(K_{t,j}\right)$ expires in the money. Then we sell one unit of the underlying and hence we end up in scenario B,

$$\begin{array}{cc} \varphi_{t+1}^{0} & \geq \varphi_{t}^{0}-D\left(t\right)\left(S_{t}^{C}-K_{t,j}\right)+D\left(t\right){\underline{S}}_{t}\\ & \geq k_{t-1,j}-\varepsilon \sigma_{t-1}+k_{t,j}-\varepsilon \\ & \geq k_{t,j}-\varepsilon \sigma_{t}. \end{array}$$

In the last inequality, we have used that $k_{t-1,j}-\varepsilon \sigma_{t-1}=x_{t-1}\geq k_{t,i}-\varepsilon \sigma_{t}$, and that $k_{t,i}=\varepsilon$.

The case where $j_{t}=i_{t}=0$ is again easy to handle, because the long and the short position cancel out and we are in scenario B at the end of the (t+1)‐st period.

Thus, after we have traded at time *u* we are either in scenario A or scenario B, which proves the assertion if $\sigma_{1}=-1$.

The proof for $\sigma_{1}=1$ is similar. We will first show that after trading at time 1 we can either be in scenario A or scenario B, and the statement of the proposition then follows by induction exactly as in the case $\sigma_{1}=-1$.

First we assume that $j_{1}>0$. Then, if the option $C_{1}\left(K_{1,j}\right)$ expires out of the money, we are in scenario A; otherwise we go short in the underlying and have

$$\begin{array}{cc} \varphi_{2}^{0} & \geq -D\left(1\right)\left(S_{1}^{C}-K_{1,j}\right)+D\left(1\right){\underline{S}}_{1}\geq k_{1,j}-\varepsilon , \end{array}$$

which corresponds to scenario B. If $j_{1}=0$, then we also have that $k_{j,1}=\varepsilon$, and hence we are in scenario B.□

According to Lemma 5.2, there is a semistatic, self‐financing trading strategy Φ for the buyer of the contract $-CVB_{u}\left(\sigma ,x,I,J\right)$, such that $\left(\varphi_{u+1}^{0},\varphi_{u+1}^{1}\right)$ only depends on $\sigma_{u},k_{u,j}$ (the investor might have some surplus in the bank account). In the following, we will use this strategy and only write $-CVB_{u}\left(\sigma_{u},k_{u,j}\right)$, respectively, ${\underline{r}}_{u}^{CVB}\left(\sigma_{u},k_{u,j}\right)$ instead of $-CVB_{u}\left(\sigma ,x,I,J\right)$, respectively, ${\underline{r}}_{u}^{CVB}\left(\sigma ,x,I,J\right)$. In the case where $\varphi_{u}^{0}\geq 0$ and $\varphi_{u}^{1}=0$, we will say that the CVB expires out of the money; otherwise we will say that it expires in the money.

The next theorem states necessary conditions for the absence of arbitrage in markets with spread‐bound ε≥0.

Theorem 5.3 Let ε≥0, s,t,u∈T such that s<t and s<u and $i\in \{0,\cdots ,N_{t}\}$, $j\in \{0,\cdots ,N_{s}\}$, $l\in \{0,\cdots ,N_{u}\}$. Fix prices as at the beginning of Section 2, with $k_{t,1}>\varepsilon$ for all t∈T. Then, for all CVBs with maturity s∈T and parameters $k_{s,j}$ and $\sigma_{s}$, the following conditions are necessary for ε‐consistency:

(i) (24) $$\begin{array}{c} \frac{{\underline{r}}_{s}^{CVB}\left(\sigma_{s},k_{s,j}\right)-{\bar{r}}_{t,i}}{\left(k_{s,j}-\varepsilon \sigma_{s}\right)-\left(k_{t,i}+\varepsilon \right)}\leq \frac{{\bar{r}}_{u,l}-{\underline{r}}_{s}^{CVB}\left(\sigma_{s},k_{s,j}\right)}{k_{u,l}+\varepsilon -\left(k_{s,j}-\varepsilon \sigma_{s}\right)},\;\text{if}\;k_{t,i}+\varepsilon <k_{s,j}-\varepsilon \sigma_{s}<k_{u,l}+\varepsilon , \end{array}$$
(ii)  
  (25) $$\begin{array}{c} \frac{{\bar{r}}_{u,l}-{\underline{r}}_{s}^{CVB}\left(\sigma_{s},k_{s,j}\right)}{k_{u,l}+\varepsilon -\left(k_{s,j}-\varepsilon \sigma_{s}\right)}\geq -1,\;\text{if}\;k_{s,j}-\varepsilon \sigma_{s}<k_{u,l}+\varepsilon , \end{array}$$
(iii)  
  (26) $$\begin{array}{cc} {\underline{r}}_{s}^{CVB}\left(\sigma_{s},k_{s,j}\right)-{\bar{r}}_{t,i} & \leq 0,\;\text{if}\;k_{s,j}-\varepsilon \sigma_{s}\geq k_{t,i}+\varepsilon , \end{array}$$
(iv)  
  (27) $$\begin{array}{c} {\underline{r}}_{s}^{CVB}\left(\sigma_{s},k_{s,j}\right)-{\bar{r}}_{t,i}=0\;\Rightarrow \;{\bar{r}}_{t,i}=0,\;\text{if}\;k_{s,j}-\varepsilon \sigma_{s}>k_{t,i}+\varepsilon . \end{array}$$

We will assume that s<t≤u and that i,l>0. Similarly, the other cases can be dealt with. In all four cases (i)–(iv), we will assume that until time *s* we followed the trading strategy described in Lemma 5.2.

(i) If (24) fails, then we set
  $$\theta =\frac{k_{u,l}+\varepsilon -\left(k_{s,j}-\varepsilon \sigma_{s}\right)}{k_{u,l}-k_{t,i}}\in \left(0,1\right)$$
  and buy $\theta C_{t}\left(K_{t,i}\right)+\left(1-\theta \right)C_{u}\left(K_{u,l}\right)-CVB_{s}\left(\sigma_{s},K_{s,j}\right)$, making an initial profit. If the calendar vertical basket $CVB_{s}\left(\sigma_{s},K_{s,j}\right)$ expires out of the money, then we have model‐independent arbitrage. Otherwise we have a short position in the underlying at time *s*. To close the short position, we buy θ units of the underlying at time *t*, and we buy 1−θ units of the underlying at time *u*. The liquidation value of this strategy at time *u* is then nonnegative,
  $$\begin{array}{cc} \left(k_{s,j}-\varepsilon \sigma_{s}+\varepsilon \right)B\left(u\right) & +\theta {\left(S_{t}^{C}-K_{t,i}\right)}^{+}\frac{B(u)}{B(t)}+\left(1-\theta \right){\left(S_{u}^{C}-K_{u,l}\right)}^{+}\\ & +\left({\underline{S}}_{s}-S_{s}^{C}\right)\frac{B(u)}{B(s)}-\theta {\bar{S}}_{t}\frac{B(u)}{B(t)}-\left(1-\theta \right){\bar{S}}_{u}\\ \geq \; & \left(k_{s,j}-\varepsilon \sigma_{s}\right)B\left(u\right)+\theta \frac{B(u)}{B(t)}\left(S_{t}^{C}-K_{t,i}-{\bar{S}}_{t}\right)+\left(1-\theta \right)\left(S_{u}^{C}-K_{u,l}-{\bar{S}}_{u}\right)\\ \geq \; & \left(k_{s,j}-\varepsilon \sigma_{s}-\theta k_{t,i}-\left(1-\theta \right)k_{u,l}-\varepsilon \right)B\left(u\right)=0. \end{array}$$
(ii) Next, assume that (25) fails. Then buying the contract
  $$C_{u}\left(K_{u,l}\right)-CVB_{s}\left(\sigma_{s},K_{s,j}\right)+k_{u,l}+\varepsilon -\left(k_{s,j}-\varepsilon \sigma_{s}\right)$$
  earns an initial profit. If $CVB_{s}\left(\sigma_{s},K_{s,j}\right)$ expires out of the money, then we leave the portfolio as it is. Otherwise we immediately enter a short position and close it at time *u*. The liquidation value is then nonnegative,
  $$\begin{array}{cc} \left(k_{s,j}-\varepsilon \sigma_{s}+\varepsilon \right)B\left(u\right) & +\left({\underline{S}}_{s}-S_{s}^{C}\right)\frac{B(u)}{B(s)}+{\left(S_{u}^{C}-K_{u,l}\right)}^{+}-{\bar{S}}_{u}\\ & +\left(k_{u,l}+\varepsilon -\left(k_{s,j}-\varepsilon \sigma_{s}\right)\right)B\left(u\right)\geq 0. \end{array}$$
(iii) If (26) fails, then we buy the contract $C_{t}\left(K_{t,i}\right)-CVB_{s}\left(\sigma_{s},k_{s,j}\right)$ for negative cost. Again we can focus on the case where $CVB_{s}\left(\sigma_{s},k_{s,j}\right)$ expires in the money. We sell one unit of the underlying at time *s* and close the short position at time *t*. The liquidation value of this strategy at time *t* is nonnegative,
  $$\left(k_{s,j}-\varepsilon \sigma_{s}+\varepsilon \right)B\left(t\right)+\left({\underline{S}}_{s}-S_{s}^{C}\right)\frac{B(t)}{B(s)}+{\left(S_{t}^{C}-K_{t,j}\right)}^{+}-{\bar{S}}_{t}\geq 0.$$
(iv) We will show that an ε‐consistent model cannot exist, if (27) fails. In every model where the probability that $CVB_{s}\left(\sigma_{s},k_{s,j}\right)$ expires in the money is zero, we could simply sell $CVB_{s}\left(\sigma_{s},k_{s,j}\right)$ and follow the trading strategy from Lemma 5.2, realizing (model‐dependent) arbitrage. On the other hand, if $CVB_{s}\left(\sigma_{s},k_{s,j}\right)$ expires in the money with positive probability, then we can use the same strategy as in the proof of (iii). At time *t*, the liquidation value of the portfolio is positive with positive probability.□

Note that, if ε=0, then $CVB_{s}\left(\sigma_{s},k_{s,j}\right)$ has the same payoff as $-C_{s}\left(K_{s,j}\right)$. Keeping this in mind, it is easy to verify that the conditions from Theorem 5.3 are a generalization of equations (4)–(6) in Cousot (2007).

It remains open whether (24)–(27) are also sufficient for the existence of an ε‐consistent model.

Conjecture 5.4 Given the conditions stated in Theorem 5.3, the given prices are ε‐consistent with the absence of arbitrage if and only if (24)–(27) hold. There is weak arbitrage whenever (24)–(26) hold but (27) fails.

Theorem 5.3 can be used to find arbitrage opportunities associated with given market prices. However, it might not be clear how to find parameters that satisfy the conditions of Definition 5.1. For the reader's convenience, we finish this section with an algorithm that can be used to create CVBs given the prices at the beginning of Section 2. It is not hard to see that it yields all possible parameter configurations. Once a particular CVB is chosen, its bid price can be obtained via (22).

(i) Pick $j_{1}\in \left\{0,\cdots ,N_{1}\right\}$ and $\sigma_{1}\in \left\{-1,1\right\}$ and set $x_{1}=k_{1,j_{1}}-\varepsilon \sigma_{1}$.
(ii) Given $\left\{x_{1},\cdots ,x_{t-1}\right\}$, $\left\{\sigma_{1},\cdots ,\sigma_{t-1}\right\}$, $\left\{j_{1},\cdots ,j_{t-1}\right\}$ and $\left\{i_{2},\cdots ,i_{t-1}\right\}$ first pick $j_{t}\in \left\{0,\cdots ,N_{t}\right\}$.
(iii) Choose $\sigma_{t}$ distinguishing the following cases:
  - if $k_{t,j_{t}}\geq x_{t-1}+\varepsilon$ set $\sigma_{t}=-1$;
  - if $k_{t,j_{t}}\leq x_{t-1}-\varepsilon$ set $\sigma_{t}=1$;
  - if $k_{t,j_{t}}=x_{t-1}$ pick $\sigma_{t}\in \left\{-1,0,1\right\}$;
  - if $k_{t,j_{t}}\in \left(x_{t-1}-\varepsilon ,x_{t-1}+\varepsilon \right)\;\setminus \;\left\{x_{t-1}\right\}$ pick $\sigma_{t}\in \left\{-1,1\right\}$.
(iv) Set $x_{t}=k_{t,j_{t}}-\sigma_{t}\varepsilon$ and pick $i_{t}\in \left\{0,\cdots ,N_{t}\right\}$ such that either $k_{t,i_{t}}\leq x_{t-1}+\sigma_{t}\varepsilon$ or $i_{t}=0$.
(v) Repeat steps (ii)–(iv).

## 6 CONCLUSION

We define the notion of ε‐consistent prices, meaning that a set of bid and ask prices for call options and the underlying can be explained by a model with bid–ask spread bounded by ε. For a single maturity, we solve the ε‐consistency problem, recovering the trichotomy consistency/weak arbitrage/model‐independent arbitrage from the frictionless case (Davis & Hobson, 2007). The interval of spread bounds for which a consistent model exists can be easily computed. The multiperiod problem seems to be rather difficult. As a first step, we provide two results: necessary explicit conditions, and equivalent semiexplicit conditions. For the latter, we invoke a recent result from Gerhold and Gülüm (2019) on approximation by peacocks. Finally, we note that section 3.3 of the Ph.D. thesis (Gülüm, 2016) discusses the multiperiod problem under simplified assumptions. In particular, it is assumed that only the underlying has a bid–ask spread, but not the options.

## ENDNOTES

1 Equations and inequalities among random variables are always understood to hold almost surely.

2 Note that we do not mention the physical probability measure, as it is of no relevance to our study.

## APPENDIX A PROOF OF THEOREM 3.1: ε‐CONSISTENCY

We first show that the conditions are necessary. Throughout the proof, we will denote the option $C_{1}\left(K_{1,i}\right)$ by $C^{i}$ to ease notation.

(i) Suppose that 1≤i<j<l are such that (13) does not hold. We buy a butterfly spread, which is the contract
  $$BF^{i,j,l}=\frac{1}{K_{j}-K_{i}}C^{i}+\frac{1}{K_{l}-K_{j}}C^{l}-\left(\frac{1}{K_{j}-K_{i}}+\frac{1}{K_{l}-K_{j}}\right)C^{j}$$
  and get an initial payment. Its payoff at maturity is positive if $S_{1}^{C}$ expires in the interval $\left(K_{i},K_{l}\right)$ and zero otherwise, and so we have model‐independent arbitrage.
  If (13) fails for i=0, we buy the contract
  $$BF^{0,j,l}=\frac{1}{K_{j}-B\varepsilon }S+\frac{1}{K_{l}-K_{j}}C^{l}-\left(\frac{1}{K_{j}-B\varepsilon }+\frac{1}{K_{l}-K_{j}}\right)C^{j}$$
  and make an initial profit. Note that *S* denotes the underlying. At maturity, the liquidation value of the contract is given by
  $$\frac{1}{K_{j}-B\varepsilon }{\underline{S}}_{1}+\frac{1}{K_{l}-K_{j}}{\left(S_{1}^{C}-K_{l}\right)}^{+}-\left(\frac{1}{K_{j}-B\varepsilon }+\frac{1}{K_{l}-K_{j}}\right){\left(S_{1}^{C}-K_{j}\right)}^{+},$$
  which is always nonnegative.
(ii) Suppose that (14) fails for 1≤i<l. Then we buy a call spread $C^{l}-C^{i}$ and invest $k_{l}-k_{i}$ in the bank account. This earns an initial profit, and at maturity the cash flow generated by the options is at least $K_{i}-K_{l}$, which means that we have arbitrage. Now we consider the case where i=0. Note that in this case (14) is equivalent to
  $$\frac{{\bar{r}}_{l}-{\underline{S}}_{0}}{k_{l}+\varepsilon }\geq -1.$$
  If this fails, we buy $C^{l}$, sell one unit of the underlying, and invest $k_{l}+\varepsilon$ in the bank account. Again we earn an initial profit, and at maturity we close the short position and have thus constructed an arbitrage strategy.
(iii) If (15) fails for 0<i<j, then we buy the call spread $C^{i}-C^{j}$ and get an initial payment. Its payoff at maturity is always nonnegative.
  If (15) fails for i=0, then we sell $C^{j}$ and buy one unit of the stock, which also yields model‐independent arbitrage.
(iv) We show that we cannot find an arbitrage‐free model for the given prices, if (16) fails. Later, in Appendix B, we will show that there is a weak arbitrage opportunity in this case (which entails, according to Definition 2.10, that there is no model‐independent arbitrage).

In any model where $P(S_{1}^{C}>K_{j})=0$, we could sell $C^{j}$. As this option is never exercised, this yields arbitrage. If, on the other hand, $P(S_{1}^{C}>K_{j})>0$ and i>0, then we buy the call spread $C^{i}-C^{j}$ at zero cost. At maturity, the probability that the options generate a positive cash flow is positive. If i=0, then we buy the contract $S-C^{j}$ instead, and at maturity the liquidation value of the portfolio is given by ${\underline{S}}_{1}-\left(S_{1}^{C}-K_{j}\right)$, which is positive with positive probability. This completes the proof of necessity.

Now we show that the conditions in Theorem 3.1 are sufficient for ε‐consistency, using Lemma 2.7. We first argue that we may w.l.o.g. assume that ${\bar{r}}_{N}={\underline{r}}_{N}=0$. Indeed, we could choose

$$k_{N+1}\geq \max\left\{\frac{{\bar{r}}_{i}k_{j}-{\underline{r}}_{j}k_{i}}{{\bar{r}}_{i}-{\underline{r}}_{j}}:\;0\leq i<j\leq N,\;{\bar{r}}_{i}-{\underline{r}}_{j}>0\right\}\vee \max\left\{k_{j}+{\underline{r}}_{j}:0\leq j\leq N\right\}$$

and set ${\bar{r}}_{N+1}={\underline{r}}_{N+1}=0$. Then all conditions from Theorem 3.1 would still hold, if we included an additional option with strike $k_{N+1}$ and bid and ask price equal to zero. So from now on we assume that ${\bar{r}}_{N}={\underline{r}}_{N}=0$.

We will first show that, for s∈{0,⋯,N}, we can find $e_{s}\in \left[{\underline{r}}_{s},{\bar{r}}_{s}\right]$ such that the linear interpolation *L* of the points $\left(k_{s},e_{s}\right),s\in \left\{0,\cdots ,N\right\}$, is convex, decreasing, and such that the right derivative of *L* satisfies $L^{\prime }\left(k_{0}\right)\geq -1$. Then we will extend *L* to a call function, and its associated measure will be the law of $D\left(1\right)S_{1}^{C}$. The sequence ${\left(e_{s}\right)}_{s\in \{1,\cdots ,N\}}$ can then be interpreted as shadow prices of the options with strikes ${\left(k_{s}\right)}_{s\in \{1,\cdots ,N\}}$.

Before we start, we will introduce some notation. For j,l∈{1,⋯,N},j<l, we denote the line connecting $\left(k_{j},{\underline{r}}_{j}\right)$ and $\left(k_{l},{\bar{r}}_{l}\right)$ by $f_{j,l}$, that is,

$$f_{j,l}\left(x\right)={\underline{r}}_{j}+\frac{{\bar{r}}_{l}-{\underline{r}}_{j}}{k_{l}-k_{j}}\cdot \left(x-k_{j}\right).$$

If $e_{s}$ is known for some s∈{0⋯,N}, then we denote the line connecting $\left(k_{s},e_{s}\right)$ and $\left(k_{i},{\bar{r}}_{i}\right),i\in \left\{s+1,\cdots ,N\right\}$ by $g_{s,i}$, that is,

$$g_{s,i}\left(x\right)=e_{s}+\frac{{\bar{r}}_{i}-e_{s}}{k_{i}-k_{s}}\cdot \left(x-k_{s}\right).$$

The linear interpolation of $\left(k_{s},e_{s}\right)$ and $\left(k_{j},{\underline{r}}_{j}\right),j\in \left\{s+1,\cdots ,N\right\}$ will be denoted by $h_{s,j}$,

$$h_{s,j}\left(x\right)=e_{s}+\frac{{\underline{r}}_{j}-e_{s}}{k_{j}-k_{s}}\cdot \left(x-k_{s}\right).$$

We will refer to the slopes of these lines as $f_{j,l}^{\prime },g_{s,i}^{\prime }$ and $h_{s,j}^{\prime }$, respectively.

First we will construct *e*_0_. To get all desired properties—this will become clear toward the end of the proof—*e*_0_ has to satisfy

(A.1) $$e_{0}\geq \max_{0\leq j<l\leq N}f_{j,l}\left(k_{0}\right),$$

and

(A.2) $$e_{0}\leq \min_{0\leq i\leq N}\left(k_{i}+{\bar{r}}_{i}-k_{0}\right).$$

We will argue that we can pick such an *e*_0_ by showing that

(A.3) $$\begin{array}{c} f_{j,l}\left(k_{0}\right)\leq k_{i}+{\bar{r}}_{i}-k_{0},\;i,j,l\in \left\{0,\cdots ,N\right\},j\leq l. \end{array}$$

Using (14) twice, we can immediately see that (A.3) holds for i≥j,

$$f_{j,l}\left(k_{0}\right)\leq {\underline{r}}_{j}+k_{j}-k_{0}\leq {\bar{r}}_{i}+k_{i}-k_{0}.$$

If, on the other hand, i<j, we rewrite the right‐hand side of (A.3) to $h_{i}\left(k_{0}\right)$, where $h_{i}\left(x\right)=-x+{\bar{r}}_{i}+k_{i}$. Then from (13), we get that

$$f_{j,l}\left(k_{i}\right)\leq {\bar{r}}_{i}=h_{i}\left(k_{i}\right),$$

and as $f_{j,l}^{\prime }\geq -1=h_{i}^{\prime }$, the inequality follows.

The above reasoning shows that existence of an *e*_0_ such that (A.1) and (A.2) hold. Next we want to construct *e*_1_ for given *e*_0_. It has to satisfy the requirements

(A.4) $$e_{1}\geq \max_{1\leq j<l\leq N}f_{j,l}\left(k_{1}\right)\vee \left(e_{0}+k_{0}-k_{1}\right)$$

and

(A.5) $$e_{1}\leq \min_{1\leq i\leq N}g_{0,i}\left(k_{1}\right).$$

Again we will argue that we can pick such an *e*_1_ by considering the corresponding inequalities. First note that the inequality

$$e_{0}+k_{0}-k_{1}\leq g_{0,i}\left(k_{1}\right),\;i\in \left\{1,\cdots ,N\right\},$$

follows directly from (A.1). Next we want to prove that

(A.6) $$f_{j,l}\left(k_{1}\right)\leq g_{0,i}\left(k_{1}\right),\;i,j,l\in \left\{1,\cdots ,N\right\},j<l.$$

Therefore, observe that

$$f_{j,l}\left(k_{0}\right)\leq e_{0}=g_{0,i}\left(k_{0}\right).$$

If i<j (A.6) follows from (13), because $f_{j,l}\left(k_{i}\right)\leq {\bar{r}}_{i}=g_{0,i}\left(k_{i}\right)$. For i=j we may simply use the fact that ${\underline{r}}_{i}\leq {\bar{r}}_{i}$ and hence we get that $f_{j,l}\left(k_{i}\right)\leq {\bar{r}}_{i}=g_{0,i}\left(k_{i}\right)$. For i>j, we may use $f_{j,l}\left(k_{0}\right)\leq e_{0}=h_{0,j}\left(k_{0}\right)$ to get

$$f_{j,l}\left(k_{1}\right)\leq h_{0,j}\left(k_{1}\right)\leq g_{0,i}\left(k_{1}\right),$$

where the last inequality follows from the fact that $h_{0,j}\left(k_{0}\right)=g_{0,i}\left(k_{0}\right)=e_{0}$ and that

$$h_{0,j}^{\prime }=\frac{{\underline{r}}_{j}-e_{0}}{k_{j}-k_{0}}\leq \frac{{\bar{r}}_{i}-e_{0}}{k_{i}-k_{0}}=g_{0,i}^{\prime }.$$

In the last step, we used that $e_{0}\geq f_{j,i}\left(k_{0}\right)$.

Now suppose we have already constructed $e_{1},\cdots e_{s-1},s\in 1,\cdots ,N$. Then for r∈{1,⋯s−1}, we have that

(A.7) $$\begin{array}{c} e_{r}\geq \left(e_{r-1}+\frac{e_{r-1}-e_{r-2}}{k_{r-1}-k_{r-2}}\cdot \left(k_{r}-k_{r-1}\right)\right)\vee \max_{r\leq j<l\leq N}f_{j,l}\left(k_{r}\right), \end{array}$$

and

(A.8) $$\begin{array}{c} e_{r}\leq \min_{r\leq i\leq N}g_{r-1,i}\left(k_{r}\right). \end{array}$$

Note that for r=1, we need an appropriate  $e_{-1}$ and $k_{-1}$ in order for (A.7) to hold. For instance, we can set $k_{-1}=-1$ and $e_{-1}=e_{0}-\left(k_{0}+1\right)\cdot \left(e_{1}-e_{0}\right)/\left(k_{1}-k_{0}\right)$.

We want to show that we can choose $e_{s}$ such that (A.7) and (A.8) hold for r=s. First, the inequality

$$e_{s-1}+\frac{e_{s-1}-e_{s-2}}{k_{s-1}-k_{s-2}}\cdot \left(k_{s}-k_{s-1}\right)\leq g_{s-1,i}\left(k_{s}\right),\;i\in \left\{s,\cdots ,N\right\},$$

is equivalent to

$$\frac{e_{s-1}-e_{s-2}}{k_{s-1}-k_{s-2}}\leq \frac{{\bar{r}}_{i}-e_{s-1}}{k_{i}-k_{s-1}},$$

which is again equivalent to

$$e_{s-1}\leq g_{s-2,i}\left(k_{s-1}\right)$$

and holds by (A.8).

The inequality,

$$f_{j,l}\left(k_{s}\right)\leq g_{s-1,i}\left(k_{s}\right),\;i,j,l\in \left\{s,\cdots ,N\right\},j<l,$$

can be shown using the same arguments as before: first we note that $f_{j,l}\left(k_{s-1}\right)\leq e_{s-1}=g_{s-1,i}\left(k_{s}\right)$ and then we distinguish between i<j, i=j and i>j.

We have now constructed a finite sequence ${\left(e_{s}\right)}_{s\in \{0,\cdots ,N\}}$. Observe that for all s∈{0,⋯,N} the bounds on $e_{s}$ from above, namely (A.1) and (A.2) for s=0,  (A.4) and (A.5) for s=1 and (A.7) and (A.8) for s>1, ensure that $e_{s}\in \left[{\underline{r}}_{s},{\bar{r}}_{s}\right]$. Denote by $L:[k_{0},k_{N}]\to R$ the linear interpolation of the points $\left(k_{s},e_{s}\right),s\in \left\{0,\cdots ,N\right\}$. Then *L* is convex, which is easily seen from

$$e_{s}\geq e_{s-1}+\frac{e_{s-1}-e_{s-2}}{k_{s-1}-k_{s-2}}\cdot \left(k_{s}-k_{s-1}\right),\;s\geq 2.$$

Furthermore, by (A.4)

$$L^{\prime }\left(k_{0}\right)=\frac{e_{1}-e_{0}}{k_{1}-k_{0}}\geq -1.$$

Finally, *L* is strictly decreasing on {L>0} that is most easily seen from $e_{s}\leq g_{s-1,N}\left(k_{s}\right)$. Therefore, *L* can be extended to a call function *R* as follows (see Proposition 2.3 in Gerhold & Gülüm, 2019):

$$\begin{array}{c} R\left(x\right)=\left\{\begin{array}{cc} L\left(k_{0}\right)+k_{0}-x, & x\leq k_{0},\\ L(x), & x\in [k_{0},k_{N}],\\ 0, & x\geq k_{N}. \end{array}\right. \end{array}$$

Let μ be the associated measure. Then $E\mu =R\left(0\right)=L\left(k_{0}\right)+k_{0}\in \left[{\underline{S}}_{0}-\varepsilon ,{\bar{S}}_{0}+\varepsilon \right]$. If $E\mu <{\underline{S}}_{0}$, we define a measure ν by setting ν(A)=μ(A−ε) for Borel sets *A*. The set A−ε is defined as {a−ε:a∈A}. Then $E\nu =E\mu +\varepsilon \in [{\underline{S}}_{0},{\bar{S}}_{0}]$. Similarly, if $E\mu >{\bar{S}}_{0}$ we define ν(A)=μ(A+ε) for Borel sets *A*, and if $E\mu \in [{\underline{S}}_{0},{\bar{S}}_{0}]$ then we simply set ν=μ. Furthermore for $x<k_{0}$, we have that $R^{\prime }\left(x\right)=-1$, therefore μ has support [ε,∞). Clearly, by definition of ν, we have that $W^{\infty }\left(\mu ,\nu \right)\leq \varepsilon$. Hence, by Lemma 2.7 the prices are ε‐consistent with the absence of arbitrage.

## APPENDIX B PROOF OF THEOREM 3.1: WEAK ARBITRAGE

As we have seen in part (iv) of the necessity proof of Theorem 3.1 (see Appendix A), there is an arbitrage opportunity that depends on the null sets of the model. We will show that there is no model‐independent arbitrage strategy. Suppose, on the contrary, that there is one. Then we can construct a portfolio $\varphi_{1}^{0}+\varphi_{1}^{1}S+\sum_{l=1}^{N}\varphi^{l}C\left(K_{l}\right)$, where $\varphi_{1}^{0},\varphi_{1}^{1},\varphi^{l}\in R$, such that its initial cost is negative, that is,

$$\varphi_{1}^{0}+\left({\left(\varphi_{1}^{1}\right)}^{+}{\bar{S}}_{0}-{\left(\varphi_{1}^{1}\right)}^{-}{\underline{S}}_{0}\right)+\sum_{l=1}^{N}\left({\left(\varphi^{l}\right)}^{+}{\bar{r}}_{l}-{\left(\varphi^{l}\right)}^{-}{\underline{r}}_{l}\right)<0,$$

and such that the liquidation value at maturity is nonnegative, that is,

$$\varphi_{1}^{0}B\left(1\right)+\left({\left(\varphi_{1}^{1}\right)}^{+}{\bar{S}}_{1}-{\left(\varphi_{1}^{1}\right)}^{-}{\underline{S}}_{1}\right)+\sum_{l=1}^{N}\varphi^{l}{\left(S_{1}^{C}-K_{l}\right)}^{+}\geq 0.$$

Without loss of generality, we can assume that $|\varphi_{1}^{0}\left|+\right|\varphi_{1}^{1}|+\sum_{l=1}^{N}\left|\varphi^{l}\right|=1$.

Next we construct $e_{0},\cdots ,e_{N}$ as in the sufficiency proof of Theorem 3.1. Clearly, we then have ${\bar{r}}_{i}=e_{i}=e_{i+1}=\cdots =e_{N}$. The idea is to consider a market with slightly different shadow prices ${\tilde{e}}_{l}$, which can be obtained from the original shadow prices $e_{l}$ by shifting them down. More precisely, we set

$$l_{0}=\max\left\{l:0\leq l\leq N,\;e_{l}+k_{l}=e_{0}+k_{0}\right\},$$

define

$$z=\min\left\{-\frac{r_{\Phi }}{2},\;\left(e_{l_{0}+1}+k_{l_{0}+1}-e_{l_{0}}-k_{l_{0}}\right)\cdot \frac{\sum_{s=l_{0}}^{N}\left(k_{s}-k_{l_{0}}\right)}{k_{l_{0}+1}-k_{l_{0}}},\;e_{N}\cdot \frac{\sum_{s=l_{0}}^{N}\left(k_{s}-k_{l_{0}}\right)}{k_{N}-k_{l_{0}}}\right\},$$

and put ${\tilde{e}}_{l}=e_{l}$ for $l\leq l_{0}$ and for $l>l_{0}$

$${\tilde{e}}_{l}=e_{l}-z\frac{k_{l}-k_{l_{0}}}{\sum_{s=l_{0}}^{N}\left(k_{s}-k_{l_{0}}\right)}.$$

Now consider a modified set of prices, where bid and ask price of the *l*th call, 0≤l≤N, are both defined by ${\tilde{e}}_{l}$. It is easy to check that these prices satisfy all conditions from Theorem 3.1, and hence do not admit any arbitrage opportunities. Indeed, the second expression in the definition of *z* guarantees that $e_{l_{0}+1}$ is not too small, that is,

$$\frac{e_{l_{0}+1}-e_{l_{0}}}{k_{l_{0}+1}-k_{l_{0}}}\geq -1,$$

and the third expression ensures that ${\tilde{e}}_{N}$ is not too small, that is, ${\tilde{e}}_{N}\geq 0$. A simple calculation shows that

$$\begin{array}{cc} \varphi_{1}^{0}+\left({\left(\varphi_{1}^{1}\right)}^{+}{\bar{S}}_{0}-{\left(\varphi_{1}^{1}\right)}^{-}{\underline{S}}_{0}\right)+\sum_{l=1}^{N}\varphi^{l}{\tilde{e}}_{l} & =\varphi_{1}^{0}+\left({\left(\varphi_{1}^{1}\right)}^{+}{\bar{S}}_{0}-{\left(\varphi_{1}^{1}\right)}^{-}{\underline{S}}_{0}\right)+\sum_{l=1}^{N}\varphi^{l}e_{l}\\ & \;-\sum_{l=l_{0}+1}^{N}\varphi^{l}\left(e_{l}-{\tilde{e}}_{l}\right)\leq r_{\Phi }-\sum_{l=l_{0}+1}^{N}\varphi^{l}\left(e_{l}-{\tilde{e}}_{l}\right)\\ & \leq r_{\Phi }+z\sum_{l=l_{0}+1}^{N}\left|\varphi^{l}\right|\frac{k_{l}-k_{l_{0}}}{\sum_{s=l_{0}}^{N}\left(k_{s}-k_{l_{0}}\right)}\\ & \leq r_{\Phi }+z\leq \frac{r_{\Phi }}{2}<0, \end{array}$$

and so the portfolio $C_{\Phi }$ in the modified market has negative cost. But its liquidation value at maturity is unchanged and hence nonnegative, and we have thus constructed a model‐independent arbitrage strategy for the modified set of prices, which is a contradiction.
